# Influence of the sFlt-1/PlGF Ratio on Clinical Decision-Making in Women with Suspected Preeclampsia

**DOI:** 10.1371/journal.pone.0156013

**Published:** 2016-05-31

**Authors:** Evelyn Klein, Dietmar Schlembach, Angela Ramoni, Elena Langer, Franz Bahlmann, Sabine Grill, Helene Schaffenrath, Reinhard van der Does, Diethelm Messinger, Wilma D. J. Verhagen-Kamerbeek, Manfred Reim, Martin Hund, Holger Stepan

**Affiliations:** 1 Department of Gynecology and Obstetrics, Klinikum Rechts der Isar, TU München, Munich, Germany; 2 Dept. of Obstetrics, University Hospital, Jena, Germany; 3 Department of Obstetrics and Gynecology, University Hospital of Innsbruck, Innsbruck, Austria; 4 Department of Obstetrics, Leipzig University, Leipzig, Germany; 5 Department of Obstetrics, Bürgerhospital, Frankfurt, Germany; 6 IST GmbH, Mannheim, Germany; 7 Medical and Scientific Affairs, Roche Diagnostics International Ltd, Rotkreuz, Switzerland; 8 Medical and Scientific Affairs, Roche Diagnostics GmbH, Penzberg, Germany; Medical Faculty, Otto-von-Guericke University Magdeburg, Medical Faculty, GERMANY

## Abstract

**Objective:**

To evaluate the influence of the soluble fms-like tyrosine kinase 1/placental growth factor ratio in physicians’ decision making in pregnant women with signs and symptoms of preeclampsia in routine clinical practice.

**Methods:**

A multicenter, prospective, open, non-interventional study enrolled pregnant women presenting with preeclampsia signs and symptoms in several European perinatal care centers. Before the soluble fms-like tyrosine kinase 1/placental growth factor ratio result was known, physicians documented intended clinical procedures using an iPad^®^ application (data locked/time stamped). After the result was available, clinical decisions were confirmed or revised and documented. An independent adjudication committee evaluated the appropriateness of decisions based on maternal/fetal outcomes. Clinician decision making with regard to hospitalization was the primary outcome.

**Results:**

In 16.9% of mothers (20/118) the hospitalization decision was changed after knowledge of the ratio. In 13 women (11.0%), the initial decision to hospitalize was changed to no hospitalization. In seven women (5.9%) the revised decision was hospitalization. All revised decisions were considered appropriate by the panel of adjudicators (McNemar test; *p* < 0.0001).

**Conclusions:**

The use of the soluble fms-like tyrosine kinase 1/placental growth factor test influenced clinical decision making towards appropriate hospitalization in a considerable proportion of women with suspected preeclampsia. This is the first study to demonstrate the impact of angiogenic biomarkers on decision making in a routine clinical practice.

## Introduction

Preeclampsia is a serious, multi-systemic disorder of pregnancy characterized by endothelial and placental dysfunction [1]. Preeclampsia affects 2–5% of all pregnancies, and is associated with maternal and fetal morbidity and mortality [2–5]. No preventive or therapeutic strategy other than aspirin or delivery is available, and the condition remains the most common reason for iatrogenic preterm delivery [6,7].

Preeclampsia is primarily a disease of the vasculature, particularly of the endothelium, and its pathology is thought to be driven by placental underperfusion, hypoxia, oxidative stress and inflammation [8–10]. An imbalance of pro-angiogenic and anti-angiogenic factors in the maternal circulation has been demonstrated during preeclampsia, specifically increased levels of the anti-angiogenic soluble fms-like tyrosine kinase 1 (sFlt-1), and reduced levels of pro-angiogenic placental growth factor (PlGF) [11,12]. A high ratio of sFlt-1/PlGF (a measure that reflects changes in both biomarkers) has been linked with preeclampsia, and has been demonstrated before its clinical onset [11,13–16].

Despite an improved understanding of preeclampsia biology, the syndrome is still defined clinically by new onset of hypertension and proteinuria after 20 weeks of gestation. As these are non-specific downstream manifestations of the syndrome and not the cause, high blood pressure or proteinuria alone have poor predictive power for preeclampsia onset and progression. There is a high unmet medical need for reliable confirmation or exclusion of diagnosis and prediction of preeclampsia in pregnant women with suspected preeclampsia; eclampsia; or hemolysis, elevated liver enzymes and low platelet count (HELLP) syndrome.

The Roche Elecsys^®^ immunoassay (Mannheim, Germany) sFlt-1/PlGF ratio is Conformité Européenne–In Vitro Diagnostics (CE-IVD) approved for use as an aid in preeclampsia diagnosis, and as an aid in short-term prediction of preeclampsia (rule-out and rule-in) in pregnant women with suspected preeclampsia in conjunction with other diagnostic and clinical information [17–20]. A case-control study of pregnant women with and without manifest preeclampsia demonstrated that an sFlt-1/PlGF ratio of 85 and above was useful in confirming the diagnosis of preeclampsia, whereas a ratio of 33 and below was useful to exclude the likelihood of imminent preeclampsia [14,17–20]. Similar cutoff values for ruling out imminent preeclampsia have been found using the Elecsys^®^ sFlt-1/PlGF immunoassay ratio [21–23].

The Preeclampsia Open Study (PreOS) is the first study to prospectively evaluate the clinical utility of the sFlt-1/PlGF test in the diagnosis of preeclampsia in pregnant women with signs and symptoms of preeclampsia in routine clinical practice. Using a study design that allowed real-time recording of decisions in the clinic, PreOS aimed to determine whether the results of the sFlt-1/PlGF test influenced clinician decision making, with regard to subsequent care.

## Methods

### Study design and patients

PreOS was a multicenter, prospective, open, non-interventional study in five centers in two countries, sponsored by Roche Diagnostics International Ltd. Study sites were selected based on familiarity with the sFlt-1/PlGF ratio, its use and interpretation, and the implications on clinical management of preeclampsia. The following ethics committees considered and approved the study protocol: Ethik-Kommission bei der Landesärztekammer Hessen (MC 170/2013; 30 July 2013); Ethikkommission der Medizinischen Universität Innsbruck (309/4.7; 13 April 2013); Ethik-Kommission Universitätsklinikum Jena (3708 02/13; 27 February 2013); Ethik-Kommission an der Medizinschen Fakultät der Universität Leipzig (053-12-23012012; 9 March 2012); and Ethikkommission, Fakultät für Medizin, Technische Universität München (5345–12; 19 April 2012). The study adhered to the Guidelines for Good Clinical Practice and the Declaration of Helsinki. Participants provided written informed consent.

The full study design and inclusion/exclusion criteria have been published previously [24]. The study included pregnant women (≥18 years of age, ≥24 weeks’ gestational age) with suspicion of preeclampsia (Table 1). Women who presented with both hypertension and proteinuria were excluded as they had a confirmed diagnosis of preeclampsia. Only women for whom determination of the sFlt-1/PlGF ratio was indicated, but not yet carried out, were included. The sFlt-1/PlGF ratio was measured only when clinically indicated as per the investigator’s judgment. During the conduct of this study, the investigators knew that the sFlt-1/PlGF ratio of 85 and above was useful in confirming the diagnosis of preeclampsia.

**Table 1:** Reasons for suspicion of preeclampsia and assessing the sFlt-1/PlGF ratio (full analysis population n = 192).

Reason n (% of full analysis population)	n = 192 (%)
New onset of elevated blood pressure	69 (35.9)
New onset of hypertension	27 (14.1)
Aggravation of pre-existing hypertension	24 (12.5)
New onset of protein in urine	29 (15.1)
New onset of proteinuria	7 (3.6)
Aggravation of pre-existing proteinuria	1 (0.5)
Other reasons for clinical suspicion of preeclampsia	164 (85.4)
Abnormal uterine Doppler ultrasound result^1^	82 (50.0)
IUGR (suspected)	49 (29.9)
Headache	37 (22.6)
Excessive edema	27 (16.5)
Epigastric pain	26 (15.9)
Severe swelling of face, hands or feet	26 (15.9)
Visual disturbances	14 (8.5)
Low platelets	14 (8.5)
Elevated liver transaminases	12 (7.3)
Sudden weight gain	4 (2.4)

IUGR, intrauterine growth restriction.

^1^ For the purpose of this study, an abnormal uterine Doppler ultrasound result was defined as: a bilateral notching in the uterine arteries and/or a mean pulsatility index >95th percentile.

The planned clinical management of pregnant women with signs and symptoms of preeclampsia was ascertained before and after knowledge of the sFlt-1/PlGF result. Initial clinical decisions for procedures (and intended procedures), based on available medical data and made prior to knowledge of the sFlt-1/PlGF test result, were entered into an electronic data capture device (iPad^®^ application) by the investigator (S1 Fig). Intended procedures included admission to hospital, induction of delivery, induction of fetal lung maturation, change in intensity of patient monitoring, and initiation or change of pharmacotherapy (S1 Table).

Entries to the iPad^®^ were time and date stamped and transferred to an online data center. The time and date of the sFlt-1/PlGF test result were recorded. Following availability of the test result, revised or confirmed clinical decisions guided by knowledge of the sFlt-1/PlGF result were documented by the investigator via the iPad^®^ application. Investigators were free in their clinical decisions (there were no recommendations based on cutoff value beyond that in the package inserts [17,18], nor were any clinical measures/procedures stipulated).

### Objectives

To assess the influence of the test on daily clinical practice, frequently used clinical decisions were chosen as primary and secondary objectives. The primary objective was to assess the influence of the sFlt-1/PlGF ratio on the physician’s decision on whether or not to hospitalize women with suspicion of preeclampsia. The primary endpoint was defined as the difference between the proportions of appropriate decisions for hospitalization/no hospitalization in women with suspicion of preeclampsia after information on the sFlt-1/PlGF test result versus the proportion of appropriate decisions before having this information available. An independent adjudication committee evaluated the clinical decisions for appropriateness based on maternal and fetal outcomes.

Secondary study objectives were exploratory. Secondary objectives reported here were key clinical decisions related to induction of delivery, induction of fetal lung maturation, and change in intensity of monitoring within one week. Decision-making endpoints were defined in a similar manner as the primary endpoint (i.e., differences between proportions of appropriate decisions). An analysis of the relationship between preeclampsia-related maternal/fetal outcomes with the sFlt-1/PlGF result was exploratory. Maternal outcomes included preeclampsia, eclampsia, HELLP syndrome, emergency hospitalization and preterm delivery. Fetal outcomes included IUGR and respiratory distress syndrome.

### Assessments

The schedule of assessments included a baseline visit, interim visits (any occasion after baseline visit when sFlt-1/PlGF was determined), a delivery visit and a visit 4 to 6 weeks postpartum. At each visit prior to delivery, clinical information, intended procedures and sFlt-1/PlGF test results were recorded (S1 Table).

### Diagnosis of preeclampsia and related outcomes

Preeclampsia was defined as the concomitant occurrence of proteinuria ≥2+ by dipstick urinalysis and elevated blood pressure (≥140 mmHg systolic and/or ≥90 mmHg diastolic, reproducible on two occasions) [24]. Diagnosis of associated conditions was protocol defined [24].

### Sample collection and measurement

Sample collection and processing were carried out according to the instructions in the package insert of the fully automated Roche Elecsys^®^ sFlt-1 and Roche Elecsys^®^ PlGF immunoassays on the **cobas e** electrochemiluminescence immunoassay platform (Roche Diagnostics Ltd. Mannheim, Germany) [15,17,18]. Measurements were performed at the investigational hospital or at a designated laboratory.

### Adjudication

Each change of the initial decision was assessed for appropriateness by two experts randomly selected from an adjudication committee comprising three independent experts who were not otherwise involved in the study; if the two experts disagreed, the third expert was consulted for a decisive opinion (blinded to the adjudications made by the other adjudicators). Adjudicators assessed the appropriateness of a change in the second decision, compared with the first decision, based on the subsequent course of the patient’s pregnancy. Adjudicators were not provided with the sFlt-1/PlGF test result. Investigators were also asked whether any new information other than the sFlt-1/PlGF ratio had become available between the two decision time points; this information was provided to adjudicators. Further details have been published previously [24].

### Patient populations

The patient populations are described in S2 Table. The definition of the per-protocol population incorporated a time limit on decisions (first decision recorded ≤96 hours before the sFlt-1/PlGF result was known and second decision recorded within 168 hours of sFlt-1/PlGF result being known). The per-protocol population was used for the analysis of the primary and key secondary endpoints. The use of time limits on decisions was implemented in the per-protocol definition to reduce the risk that other factors could have influenced physicians’ decisions in this population. For statistical analyses where clinical decisions did not play a role, the full analysis population or the safety population were used, as appropriate.

### Statistical analysis

The sample size was based on the primary endpoint: 143 evaluable participants were necessary to detect (with 90% power and a two-sided McNemar test [alpha = 0.05]) a difference of 15% between the proportion of appropriate changes and the proportion of inappropriate changes, assuming that the percentage of discordant decisions was 30% in the analysis population. Assuming that not all participants would be evaluable, at least 150 participants were required. The proportion of appropriate and inappropriate changes was compared using a two-sided exact McNemar test at a significance level of 0.05.

Exploratory logistic regression analyses were performed to investigate the impact of various factors (assumed as fixed effects) on the endpoint of investigator decision regarding hospitalization after measurement of the sFlt-1/PlGF ratio at the baseline visit. First, univariate logistic regression was performed for each factor. In the next step, multiple logistic regressions included all factors with a significance level of 0.25 in the univariate analysis. Finally, multiple logistic regression analysis using a backward elimination procedure was performed with a significance level of 0.05 for explanatory factors to stay in the final model. The Hosmer and Lemeshow goodness-of-fit test was applied for each model. Only models with a *p* > 0.2 were accepted. The linear relationship between any continuous explanatory variable and logit transformation of the dependent variable was checked with graphical methods. The outcomes were examined according to sFlt-1/PlGF level, using the categories <33, 33–<85, and ≥85.

## Results

### Study population

**Fig 1:**
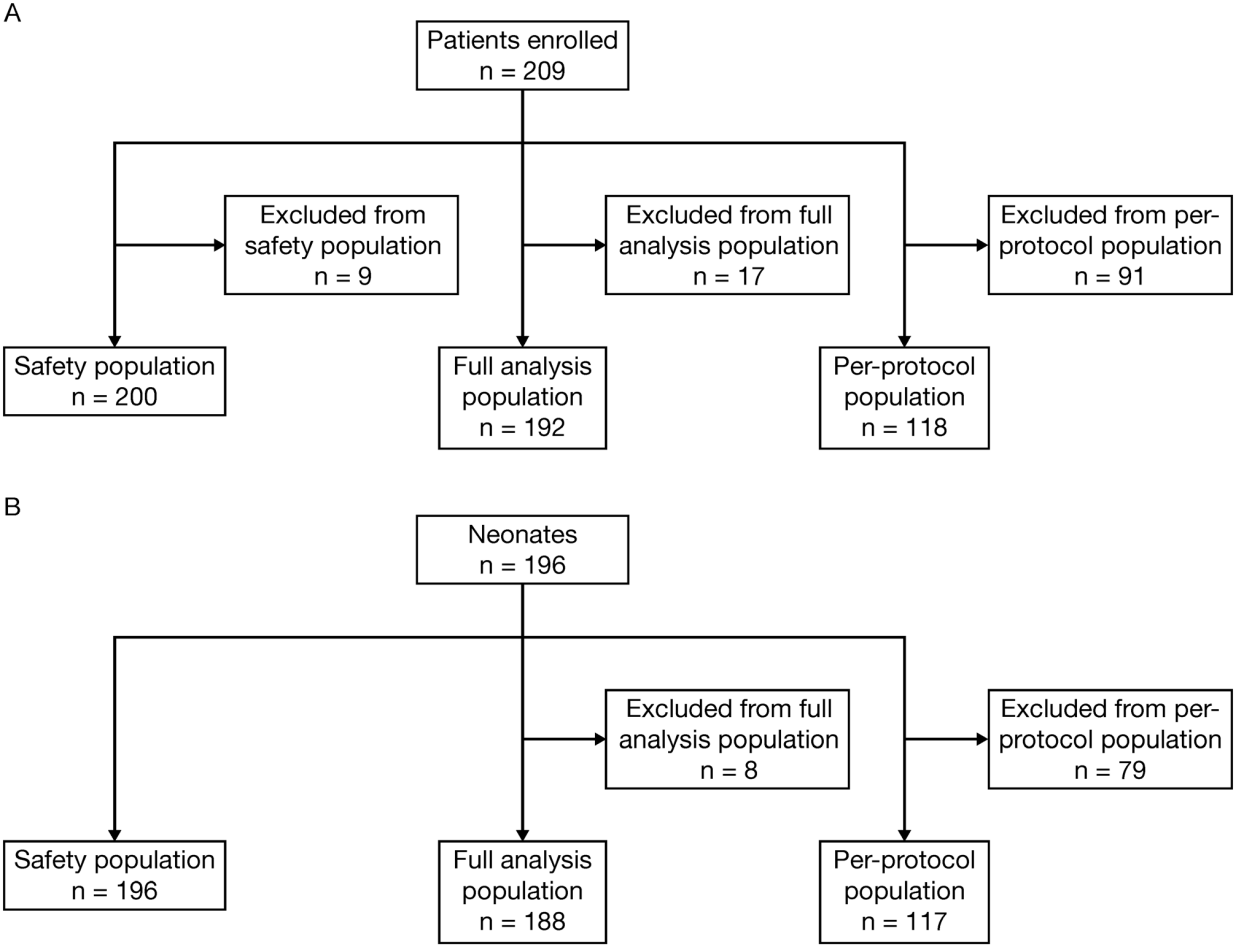
Patient populations in the PreOS study for (A) mothers and (B) neonates.

The study enrolled 209 women from 5 hospitals (Fig 1A; S3 Table). A total of 200 mothers attended the baseline visit, 11 had an interim visit, 2 had a second interim visit, 194 had a delivery visit and 158 had a postpartum visit (S4 Table). A total of 192 mothers were included in the full analysis population, and 118 were included in the per-protocol population. Reasons for exclusion from the per-protocol population are listed in S5 Table, and these included instances where the decision on hospitalization was made >96 hours before knowledge of the sFlt-1/PlGF result or >168 hours after knowledge of the sFlt-1/PlGF result. The exclusion of patients from the per-protocol population did not lead to any major differences in the baseline characteristics between this population, the full analysis population and the safety population. The safety populations and neonate populations are shown in Fig 1A and 1B.

Abnormal uterine perfusion and new onset of elevated blood pressure were the most common reasons for suspicion of preeclampsia for the full analysis population (Table 1). In the safety population, the mean number of pregnancies (including current) was 2.1 (range, 1–8), and 45.5% of women were primipara. In five women (2.5%), there was a multiple-gestation pregnancy (two fetuses each). The mean gestational age at the baseline visit was week 32+4. Pregnancy characteristics in the per-protocol population were comparable. Patient demographics are reported in Table 2. In the safety population, 7.5% of women had been diagnosed with preeclampsia during a previous pregnancy.

**Table 2:** PreOS patient demographics and baseline characteristics.

Characteristics	Per-protocol population n = 118	Safety population n = 200	Full analysis population n = 192
**Age (years)**			
Median (min–max)	31.0 (20–43)	31.1 (19–45)	31.2 (19–45)
**Height (cm)**			
Median (min–max)	166.0 (150–183)	166.0 (150–183)	166.5 (150–183)
**Pre-pregnancy weight (kg)**			
Median (min–max)	67.0 (60–79)	67.0 (42–170)	72.7 (42–170)
**Body mass index (kg/m**^**2**^**)**			
Median (min–max)	23.8 (22–29)	23.8 (17–60)	26.2 (17–60)
**Race, n (%)**			
European/White	117 (99.2%)	198 (99.0%)	190 (99.0%)
African/Black	1 (0.8%)	1 (0.5%)	1 (0.5%)
Asian	–	1 (0.5%)	1 (0.5%)
**Ethnicity, n (%)**			
Hispanic/Latino	1 (0.8%)	3 (1.5%)	3 (1.6%)
Non-Hispanic/non-Latino	113 (95.8%)	192 (96.0%)	184 (95.8%)
Unknown	4 (3.4%)	5 (2.5%)	5 (2.6%)
**Preeclampsia diagnosed in previous pregnancies, n (%)**			
Yes	10 (8.5%)	15 (7.5%)	14 (7.3%)
No	108 (91.5%)	185 (92.5%)	178 (92.7%)
**Eclampsia diagnosed in previous pregnancies, n (%)**			
Yes	1 (0.8%)	2 (1.0%)	2 (1.0%)
No	117 (99.2%)	198 (99.0%)	190 (99.0%)
**HELLP syndrome diagnosed in previous pregnancies, n (%)**			
Yes	6 (5.1%)	8 (4.0%)	7 (3.6%)
No	110 (93.2%)	190 (95.0%)	183 (95.3%)
Unknown	2 (1.7%)	2 (1.0%)	2 (1.0%)
**IUGR diagnosed in previous pregnancies, n (%)**			
Yes	2 (1.7%)	3 (1.5%)	3 (1.6%)
No	114 (96.6%)	194 (97.0%)	186 (96.9%)
Unknown	2 (1.7%)	3 (1.5%)	3 (1.6%)
**Family history of preeclampsia (mother or sister), n (%)**			
Yes	1 (0.8%)	1 (0.5%)	1 (0.5%)
No	87 (73.7%)	148 (74.0%)	141 (73.4%)
Unknown	30 (25.4%)	51 (25.5%)	50 (26.0%)

HELLP, indicates hemolysis, elevated liver enzymes and low platelets; IUGR, intrauterine growth restriction; PreOS, Preeclampsia Open Study.

### sFlt-1/PlGF testing (per-protocol population)

Intended procedures before knowledge of the sFlt-1/PlGF result were recorded on average 9.5 hours (median, 1.7; range, 0.1–92.4) before the sFlt-1/PlGF ratio was known. Revised or confirmed procedures were recorded on average 20.6 hours (median, 4.1; range, <0.1–163.9) after the sFlt-1/PlGF result was known.

### Primary endpoint: decision to hospitalize (per-protocol population)

**Fig 2:**
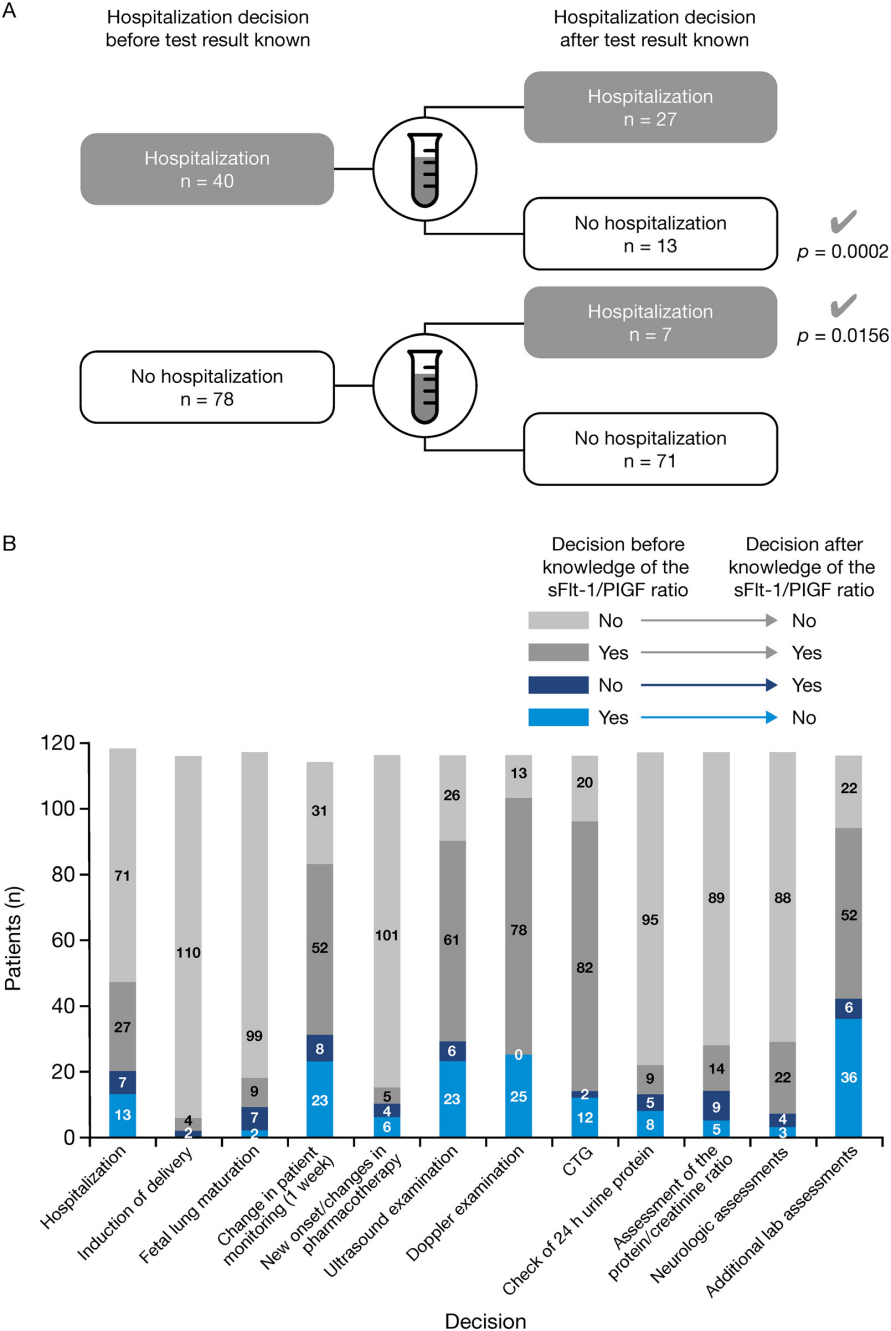
(A) Primary endpoint decisions on hospitalization changes based on the sFlt-1/PlGF result (per-protocol population, n = 118) and (B) summary of all endpoint decisions before and after knowledge of the sFlt-1/PlGF ratio (all changes were considered appropriate by the adjudicators) (per-protocol population, n = 118).

In 98 women (83.1%), investigators made no changes to their initial decision after seeing the sFlt-1/PlGF ratio result. There were 20 changes (16.9%) regarding the decision hospitalization/no hospitalization (Table 3). The primary endpoint decisions on hospitalization changes are shown in Fig 2A. In 13 mothers (11.0%), the decision was revised from ‘hospitalization’ to ‘no hospitalization’, two of whom (one with an sFlt-1/PlGF ratio of 305) went on to develop preeclampsia. In seven mothers (5.9%), the revised decision was ‘hospitalization’; four of these women went on to develop preeclampsia. Of these seven mothers, four had an sFlt-1/PlGF ratio of more than 85 (two had additional risk factors and were subsequently diagnosed with preeclampsia and two [one of whom had an additional risk factor] did not develop preeclampsia). A fifth patient had an sFlt-1/PlGF ratio of 60.2 (and no other risk factors) and developed preeclampsia. The two remaining women with a revised decision to hospitalize had a ratio below 33 (both with other risk factors): one developed preeclampsia and the other did not. In both of these women with an sFlt-1/PlGF ratio below 33 the further course of their pregnancies was uneventful. The newborn of the patient with preeclampsia was small for gestational age but otherwise healthy.

**Table 3:** Analysis of decisions after sFlt-1/PlGF ratio measurement (per-protocol population, n = 118).

Endpoint	Intended procedure before knowledge of sFlt-1/PlGF ratio	Intended procedure after knowledge of sFlt-1/PlGF ratio	n (%)	n (%) of appropriate changes	McNemar test (exact), *p* value	Median gestational age at change of decision, weeks+days	Median gestational age at delivery, weeks+days
Primary endpoint: hospitalization (n = 118)	Yes	Yes	27 (22.9%)	–	–	–	–
	No	No	71 (60.2%)	–	–	–	–
	Yes	No	13 (11.0%)	13 appropriate (100.0)	0.0002	35+0	38+5
	No	Yes	7 (5.9%)	7 appropriate (100.0)	0.0156	32+4	35+6
	Any change	Any change	20 (16.9%)	20 appropriate (100.0)	<0.0001	34+2	37+6
Secondary endpoint: induction of delivery (n = 116*)	Yes	Yes	4 (3.4%)	–	–	–	–
	No	No	110 (94.8%)	–	–	–	–
	No	Yes	2 (1.7%)	2 appropriate (100.0)	0.5000	37+6	38+4
	Any change	Any change	2 (1.7%)	2 appropriate (100.0)	0.5000	37+6	38+4
Secondary endpoint: induction of fetal lung maturation (n = 117*)	Yes	Yes	9 (7.7%)	–	–	–	–
	No	No	99 (84.6%)	–	–	–	–
	Yes	No	2 (1.7%)	2 appropriate (100.0)	0.5000	30+0	33+6
	No	Yes	7 (6.0%)	7 appropriate (100.0)	0.0156	29+5	32+0
	Any change	Any change	9 (7.7%)	9 (100.0)	0.0039	29+5	32+0
Secondary endpoint: change of patient monitoring intensity within one week (n = 114*)	Yes	Yes	52 (45.6%)	–	–	–	–
	No	No	31 (27.2%)	–	–	–	–
	Yes	No	23 (20.2%)	23 appropriate (100.0)	<0.0001	33+6	38+4
	No	Yes	8 (7.0%)	8 appropriate (100.0)	0.0078	30+6	37+3
	Any change	Any change	31 (27.2%)	31 appropriate (100.0)	<0.0001	33+4	38+4

PlGF, placental growth factor; sFlt-1, soluble fms-like tyrosine kinase 1.

* Data missing.

All changes in hospitalization decisions were considered appropriate by the adjudicators, who were blinded to the sFlt-1/PlGF ratio (Table 3). There were only four mothers where the two adjudicators did not agree initially, requiring the use of a third adjudicator. The McNemar test on the appropriateness of the alterations in decisions was statistically significant (*p* < 0.0001) for the analysis of the primary endpoint, i.e., any change in the decision of hospitalization. The McNemar test was also significant for change from ‘hospitalization’ to ‘no hospitalization’ (*p* < 0.0002) as well as change from ‘no hospitalization’ to ‘hospitalization’ (*p* = 0.0156).

When the decision was changed from ‘hospitalization’ to ‘no hospitalization’ (13 women), the median sFlt-1/PlGF ratio was 11.8 at a median gestational age of 34 weeks (range, week 27+2 to week 38+5) at the time of application of the test. The median gestational age at delivery was 3 weeks + 5 days after the median gestational age at decision. In these women, the median delivery time was week 38+5 (range, week 31+5 to week 40+0) (Table 3). Twenty-three clinical events were recorded in these 13 women, the majority of which were induced delivery, preterm delivery or Cesarean delivery.

When the decision was changed from ‘no hospitalization’ to ‘hospitalization’ (7 women), the median sFlt-1/PlGF ratio was 92.6. There was a difference of 3 weeks + 2 days between the median gestational age at decision and the median gestational age at delivery (women were not necessarily hospitalized for the whole time between change of decision and delivery). The median gestational age at delivery was week 35+6 (range, week 32+0 to week 39+6) (Table 3).

### Secondary endpoints: other clinical decisions

For 98.2% of women (114/116), the physician’s decision to induce delivery was unchanged after the sFlt-1/PlGF test. There were only two changes to the decision to deliver, where the physician decided to induce delivery after interpretation of the test result (Table 3). These changes were deemed appropriate by the adjudication committee. There were nine changes in decisions regarding management of fetal lung maturation. In seven women, the sFlt-1/PlGF test influenced the physician to induce fetal lung maturation, and in two women, the decision was revised to not induce fetal lung maturation.

No change in intensity of patient monitoring within one week was intended at both decision time points in 31 women. In eight women, monitoring intensity was decided to be changed after the sFlt-1/PlGF result. Importantly, after the sFlt-1/PlGF result no change in intensity of monitoring was planned for 23 women for whom a change had initially been intended (Table 3). All revised decisions were assessed to be appropriate. A summary of all primary and secondary endpoint decision changes is shown in Fig 2B.

### Intended procedures and outcomes

Analysis of intended procedures (hospitalization/induction of delivery/induction of fetal lung maturation) versus outcomes is presented in Table 4. Overall, 24 of 118 women (20.3%) in the per-protocol population and 36 of 183 women (19.7%) in the full analysis population evaluable for the occurrence of preeclampsia were diagnosed with preeclampsia at some point during the study. The proportion of women developing preeclampsia was highest in the subgroup of patients with a change in decision from ‘no hospitalization’ to ‘hospitalization’ (4/7 developed preeclampsia [57.1%]). This proportion was considerably higher than in women with a change in decision from ‘hospitalization’ to ‘no hospitalization’ (2/13 developed preeclampsia [15.4%]). Women whose intended procedure changed from ‘hospitalization’ to ‘no hospitalization’ had no higher risk of developing preeclampsia than women who were not planned to be hospitalized at both decision time points (11/71 [15.5%]).

**Table 4:** Intended procedures before and after knowledge of the sFlt-1/PlGF ratio and selected outcomes for mothers/neonates (per-protocol population, n = 118).

Outcomes	Intended procedure	Before knowledge of sFlt-1/PlGF ratio	After knowledge of sFlt-1/PlGF ratio	n (%)	Outcome, yes n (%)	Outcome, no n (%)	Missing n (%)	Median gestational age at outcome (if yes), weeks+days
**Preeclampsia/eclampsia/HELLP syndrome**	Admission to hospital	Yes	Yes	27 (22.9)	7 (25.9)	20 (74.1)	–	32+5
		No	Yes	7 (5.9)	4 (57.1)	3 (42.9)	–	32+4
		Yes	No	13 (11.0)	2 (15.4)	11 (84.6)	–	34+0
		No	No	71 (60.2)	11 (15.5)	60 (84.5)	–	35+4
	Induction of delivery	Yes	Yes	4 (3.4)	1 (25.0)	3 (75.0)	–	38+4
		No	Yes	2 (1.7)	–	2 (100.0)	–	–
		No	No	110 (93.2)	23 (20.9)	87 (79.1)	–	32+5
		Missing	No	1 (0.8)	–	1 (100.0)	–	–
		No	Missing	1 (0.8)	–	1 (100.0)	–	–
	Induction of fetal lung maturation	Yes	Yes	9 (7.6)	3 (33.3)	6 (66.7)	–	29+4
		No	Yes	7 (5.9)	5 (71.4)	2 (28.6)	–	30+2
		Yes	No	2 (1.7)	1 (50.0)	1 (50.0)	–	29+1
		No	No	99 (83.9)	15 (15.2)	84 (84.8)	–	35+5
		No	Missing	1 (0.8)	–	1 (100.0)	–	–
**Emergency hospitalization**	Admission to hospital	Yes	Yes	27 (22.9)	5 (18.5)	22 (81.5)	–	29+4
		No	Yes	7 (5.9)	3 (42.9)	4 (57.1)	–	29+2
		Yes	No	13 (11.0)	2 (15.4)	11 (84.6)	–	32+3
		No	No	71 (60.2)	13 (18.3)	58 (81.7)	–	36+6
	Induction of delivery	Yes	Yes	4 (3.4)	1 (25.0)	3 (75.0)	–	38+4
		No	Yes	2 (1.7)	–	2 (100.0)	–	–
		No	No	110 (93.2)	20 (18.2)	90 (81.8)	–	35+6
		Missing	No	1 (0.8)	1 (100.0)	–	–	25+6
		No	Missing	1 (0.8)	1 (100.0)	–	–	35+7
	Induction of fetal lung maturation	Yes	Yes	9 (7.6)	3 (33.3)	6 (66.7%)	–	29+0
		No	Yes	7 (5.9)	2 (28.6)	5 (71.4%)	–	32+1
		Yes	No	2 (1.7)	1 (50.0)	1 (50.0%)	–	28+1
		No	No	99 (83.9)	16 (16.2)	83 (83.8%)	–	37+3
		No	Missing	1 (0.8)	1 (100.0)	–	–	35+5
**Preterm delivery**	Admission to hospital	Yes	Yes	27 (22.9)	12 (44.4)	15 (55.6)	–	32+6
		No	Yes	7 (5.9)	5 (71.4)	2 (28.6)	–	35+3
		Yes	No	13 (11.0)	2 (15.4)	11 (84.6)	–	34+2
		No	No	71 (60.2)	13 (18.3)	58 (81.7)	–	31+3
	Induction of delivery	Yes	Yes	4 (3.4)	–	4 (100.0)	–	–
		No	Yes	2 (1.7)	1 (50.0)	1 (50.0)	–	36+6
		No	No	110 (93.2)	29 (26.4)	81 (73.6)	–	32+3
		Missing	No	1 (0.8)	1 (100.0)	–	–	35+3
		No	Missing	1 (0.8)	1 (100.0)	–	–	36+6
	Induction of fetal lung maturation	Yes	Yes	9 (7.6)	7 (77.8)	2 (22.2)	–	33+3
		No	Yes	7 (5.9)	6 (85.7)	1 (14.3)	–	31+4
		Yes	No	2 (1.7)	2 (100.0)	–	–	33+6
		No	No	99 (83.9)	16 (16.2)	83 (83.8)	–	35+3
		No	Missing	1 (0.8)	1 (100.0)	–	–	36+6
**IUGR (fetus)**	Admission to hospital	Yes	Yes	27 (23.1)	6 (22.2)	20 (74.1)	1 (3.7)	30+4
		No	Yes	7 (6.0)	3 (42.9)	4 (57.1)	–	29+6
		Yes	No	13 (11.1)	2 (15.4)	10 (76.9)	1 (7.7)	32+0
		No	No	70 (59.8)	15 (21.4)	55 (78.6)	–	34+3
	Induction of delivery	Yes	Yes	4 (3.4)	1 (25.0)	3 (75.0)	–	36+4
		No	Yes	2 (1.7)	1 (50.0)	1 (50.0)	–	37+5
		No	No	109 (93.2)	24 (22.0)	83 (76.1)	2 (1.8)	31+6
		Missing	No	1 (0.9)	–	1 (100.0)	–	–
		No	Missing	1 (0.9)	–	1 (100.0)	–	–
	Induction of fetal lung maturation	Yes	Yes	9 (7.7)	3 (33.3)	6 (66.7)	–	32+5
		No	Yes	7 (6.0)	4 (57.1)	2 (28.6)	1 (14.3)	28+6
		Yes	No	2 (1.7)	2 (100.0)	–	–	29+4
		No	No	98 (83.8)	17 (17.3)	80 (81.6)	1 (1.0)	34+2
		No	Missing	1 (0.9)	–	1 (100.0)	–	–
**RDS (neonate)**	Admission to hospital	Yes	Yes	27 (23.1)	9 (33.3)	18 (66.7)	–	31+2
		No	Yes	7 (6.0)	2 (28.6)	5 (71.4)	–	33+4
		Yes	No	13 (11.1)	1 (7.7)	12 (92.3)	–	31+5
		No	No	70 (59.8)	7 (10.0)	63 (90.0)	–	29+1
	Induction of delivery	Yes	Yes	4 (3.4)	–	4 (100.0)	–	–
		No	Yes	2 (1.7)	–	2 (100.0)	–	–
		No	No	109 (93.2)	19 (17.4)	90 (82.6)	–	30+6
		Missing	No	1 (0.9)	–	1 (100.0)	–	–
		No	Missing	1 (0.9)	–	1 (100.0)	–	–
	Induction of fetal lung maturation	Yes	Yes	9 (7.7)	6 (66.7)	3 (33.3)	–	32+1
		No	Yes	7 (6.0)	6 (85.7)	1 (14.3)	–	31+5
		Yes	No	2 (1.7)	1 (50.0)	1 (50.0)	–	31+5
		No	No	98 (83.8)	6 (6.1)	92 (93.9)	–	29+3
		No	Missing	1 (0.9)	–	1 (100.0)	–	–

HELLP, hemolysis, elevated liver enzymes and low platelet count; IUGR, intrauterine growth restriction; PlGF, placental growth factor; RDS, respiratory distress syndrome; sFlt-1, soluble fms-like tyrosine kinase 1.

Out of 84 per-protocol patients with a second decision of ‘no hospitalization’, there were 13 patients (15.5%) with the outcome preeclampsia/eclampsia/HELLP syndrome (11 patients with no/no and two with yes/no decisions as to hospitalization [all patients had preeclampsia, only one developed HELLP syndrome, and there were no cases of eclampsia]) (Table 4). In the woman who developed HELLP syndrome, the first decision of ‘no hospitalization’ remained ‘no hospitalization’ despite an sFlt-1/PlGF ratio of 242.6 (far above the 85 cutoff value). Of the 13 women with the composite endpoint mentioned above, seven had a first diagnosis of preeclampsia more than 14 days after the sFlt-1/PlGF test result. One woman had preeclampsia 14 days after an sFlt-1/PlGF ratio of 63.8. Therefore, there were only two cases of preeclampsia within 14 days of the test for which the mothers had an sFlt-1/PlGF ratio below 33. For both of these women, apart from the diagnosis of preeclampsia, no further clinically untoward events were reported for mother or newborn.

Higher proportions of women with preeclampsia were also found in those where no induction of fetal lung maturation was changed to induction with the sFlt-1/PlGF test result being available (5/7 [71.4%]). There were 23 women (19.5%) who required emergency hospitalization due to preeclampsia.

### Exploratory analysis: relationship between preeclampsia-related outcomes and the sFlt-1/PlGF ratio

Exploratory analyses using multiple logistic regression with backward elimination showed that the risk for clinically important outcomes (emergency hospitalization and preterm delivery) in women with an sFlt-1/PlGF result of below 33 was lower than that for women with an sFlt-1/PlGF result of 85 and above (S6 Table). The risk for clinically important outcomes (e.g., IUGR and respiratory distress syndrome) in the fetus/neonate was also lower if the sFlt-1/PlGF result was below 33, compared with 85 and above.

### Exploratory analysis: relationship between intended hospitalization rates and the sFlt-1/PlGF ratio

The intended hospitalization rates before knowledge of the sFlt-1/PlGF ratio increased by 9.5% after knowledge of the sFlt-1/PlGF ratio in women with an sFlt-1/PlGF ratio 85 and above. The intended hospitalization rate decreased by 10.7% after knowledge of the sFlt-1/PlGF ratio in women with an sFlt-1/PlGF ratio below 33 (S7 Table).

### sFlt-1/PlGF and outcomes

Preeclampsia occurred most often in patients with an sFlt-1/PlGF ratio of ≥85 (40.5%), compared with 33–<85 (28.1%) and <33 (9.8%). It is notable that, in the safety population of 200 women, the only cases of eclampsia (n = 2), HELLP syndrome (n = 3), pulmonary edema (n = 1), acute renal failure (n = 3) and cerebral hemorrhage (n = 1) occurred in patients with an sFlt-1/PlGF ratio of 85 or above.

A total of 75 per-protocol women had an sFlt1/PlGF ratio below 33. Of these, seven patients (9.3%) had a subsequent diagnosis of preeclampsia. In two of these women, preeclampsia was diagnosed far beyond 14 days after the test (at 58 and 73 days post-test). None of the patients with an sFlt-1/PlGF ratio below 33 who developed preeclampsia developed eclampsia, HELLP syndrome, or experienced any other clinical important negative outcome for the mother or the neonate, with the exception of one case of a small-for-gestational-age baby.

### Safety

There were no reports of incidents or indirect harm associated with the use of the sFlt-1/PlGF test in routine clinical practice in this population with suspected preeclampsia.

## Discussion

Among our study that included more than 200 pregnant women, only one in five women for whom physicians suspected preeclampsia actually went on to develop preeclampsia. This indicates that in routine clinical practice, preeclampsia may be over-diagnosed and suspected preeclampsia may be over treated [10,25].

For most women, investigators made no changes to their initial clinical-management decision after knowing the result of the sFlt-1/PlGF test. Nonetheless, planned changes to the first decision upon knowledge of the test result, most notably regarding hospitalization, were observed in a clinically relevant proportion of women. Seven women, who would otherwise have been sent home, were hospitalized after the test result had become known.

Changed decisions regarding hospitalization were generally in agreement with the risk of the patient developing a major clinical event such as preeclampsia or undergoing preterm delivery. The proportion of women developing preeclampsia was highest in the group of women who were initially not intended to be hospitalized, but who were then planned for hospitalization after the test result was available. Notably, this percentage was more than twice that in patients planned to be hospitalized at both decision time points and almost four times higher than in patients with a second decision of no hospitalization, regardless of whether the initial decision had or had not been hospitalization. This confirms that, generally, the women at highest risk–the ‘right’ women–were hospitalized after the sFlt-1/PlGF test. In 13 women, the initial decision of ‘hospitalize’ was changed to ‘no hospitalization’ after the sFlt-1/PlGF test. In two of these mothers preeclampsia was diagnosed, with one having an sFlt-1/PlGF ratio of 305.3. Both women had no major complications during the further course of their pregnancies.

Changed decisions regarding hospitalization concurred with important diagnoses in fetuses and neonates, such as IUGR and respiratory distress syndrome. The appropriateness of decisions was confirmed by adjudication, and women could be safely identified for planned ‘step-down’ management according to their sFlt-1/PlGF result. This was the case with respect to planned hospitalization in 11.0% of all women in the per-protocol population (13/118). There was also step-down management intended for the use of other clinical and laboratory investigations. A decision to step-up management in terms of hospitalization was made for 5.9% of women (7/118), resulting in better targeted use of hospital services and care to at-risk population in an appropriate setting. Most importantly, among initially planned hospitalizations, 32.5% (13/40) were identified as not being necessary in the short term, which has clear economic implications. Appropriate and timely decisions on hospitalization may also have helped to reduce emergency hospitalization rates.

In this study, the risk for preeclampsia-related maternal and fetal outcomes increased along with increasing sFlt-1/PlGF ratios and was highest in women with an sFlt-1/PlGF ratio of 85 and above. This is in line with previously published data showing that in women with suspected preeclampsia an sFlt-1/PlGF ratio of 85 and above predicted adverse outcomes occurring within 2 weeks [26].

A limitation of the study was the large number of women who were excluded from the per-protocol analysis due to delayed reporting of the decision on the iPad^®^ after knowledge of the ratio, and outcomes (preeclampsia/delivery) taking place before the intended procedures were recorded. It is important to note that the study data were generated using the Elecsys^®^ immunoassay sFlt-1/PlGF ratio cutoff of 85, but the optimal cutoff ratio to aid diagnosis of preeclampsia may differ when other assays are used. Furthermore, as the sites involved in this study had familiarity with the sFlt-1/PlGF test and its use, as well as substantial expertise in management of preeclampsia, this may limit the generalizability of the results. Although the sFlt-1/PlGF ratio has greater diagnostic accuracy compared with current standard of care, the cutoff values and reference ranges for the test only apply to singleton pregnancies, and so the results should be interpreted with caution in women with multiple pregnancies. The sFlt-1/PlGF ratio has not been evaluated as a universal screening test, and is not intended to replace other techniques for monitoring patients at risk of preeclampsia; the test should only be offered to women who are considered at high risk of preeclampsia, and should be used in the context of other established diagnostic tools. Finally, the appropriate level of monitoring of women with an intermediate sFlt-1/PlGF ratio (38–85 [early-onset preeclampsia] or 38–110 [late-onset]), including the timing interval for follow-up testing and clinical consultation, remains to be ascertained.

PreOS is the first study that examined the influence of a test result on clinical decision making applied to the diagnosis of preeclampsia. The study has several strengths, including the use of the iPad^®^ application. This documentation method was chosen to allow results to be recorded in real time and in a consistent manner between investigators and study hospitals. The use of an adjudication process was also important as this reduced bias and provided confidence that changed decisions as a result of the sFlt-1/PlGF ratio did not negatively impact the patient. The reliability of the sFlt-1/PlGF ratio in this regard is also shown by the fact that all four patients of the safety population who developed eclampsia, HELLP syndrome or both had been detected as having an increased risk by a markedly elevated sFlt-1/PlGF ratio before the clinical diagnosis of preeclampsia could be established. Finally, PreOS was a multicenter study and the data collected reflect the real-world clinical practice use of the sFlt-1/PlGF test in clinical decision making in different hospitals operating according to their local procedures and processes.

Recently, a prospective study of prediction of preeclampsia in women with suspicion of preeclampsia (PROGNOSIS; Prediction of Short-Term Outcome in Pregnant Women with Suspected Preeclampsia Study) has reported excellent ability of an sFlt-1/PlGF ratio of 38 and below to predict absence of preeclampsia within the next week (negative predictive value of 99.3% (95% confidence interval [CI]: 97.9–99.9) and good capacity of an sFlt-1/PlGF ratio above 38 to rule in preeclampsia within the next four weeks (36.7% positive predictive value (95% CI: 28.4–45.7) [27]. Ongoing studies are examining how this new cutoff value (for prediction, rather than diagnosis) has the potential to influence clinical practice. A recent study has suggested that low PlGF is predictive for preeclampsia within 14 days in women <35 weeks’ gestation [28]. However, the PROGNOSIS study, which also evaluated the predictive value of using either sFlt-1 or PlGF alone, did not show a superior predictive power for these single biomarkers compared with the ratio [27].

This study was non-interventional in its approach; further research should be performed to confirm the clinical utility of the sFlt-1/PlGF ratio indicated by our observational data. One potential avenue for further study could be a randomized, controlled trial in which one group of investigators are blinded to the test result to evaluate whether availability of the sFlt-1/PlGF ratio has an impact on clinical outcomes, including prolongation of the pregnancy, and socio-economic factors.

The reduction of inappropriate hospital admissions is an important consideration in order to avoid unnecessary stress and anxiety for the patient, and to reduce the financial burden for the healthcare provider. The wider adoption of the sFlt-1/PlGF ratio in maternity care could support the targeted delivery of clinical care by helping to identify those women who are at high risk of developing preeclampsia, and who therefore should be prioritized for further investigation and management, while those who are deemed low risk can be reassured and avoid unnecessary hospitalization.

## Conclusions

PreOS is the first study to demonstrate the impact of angiogenic biomarkers (in this case, sFlt-1/PlGF) on physicians’ clinical decision making for pregnant women with suspicion of preeclampsia in a routine clinical setting. The sFlt-1/PlGF ratio has the potential to be implemented in clinical practice to guide appropriate intensity of patient management with respect to hospitalization and therapeutic decisions in a clinically relevant proportion of pregnant women with signs and symptoms of preeclampsia, eclampsia or HELLP syndrome.

## Supporting Information

S1 Fig iPad^®^ application used in PreOS. (DOCX)

S1 Table Schedule of assessments in PreOS* [1]. IUGR, intrauterine growth restriction; PlGF, placental growth factor; PreOS, Preeclampsia Open Study^1^; sFlt-1, soluble fms-like tyrosine kinase 1.* Measures listed not stipulated by the protocol. Documentation of data as available based on routine proceedings at the center and clinical judgment. † Intended procedures included: admission to hospital; induction of delivery; induction of fetal lung maturity; time interval to next visit; change in intensity of patient monitoring (intervals of blood pressure measurement; blood pressure home monitoring); assessment of protein in urine; new onset or changes in (pharmaco-)therapy for the indication; ultrasound study (for IUGR, amniotic fluid volume) and/or uterine artery Doppler (for pulsatility index notching); cardiotocography; 24-hour urinary protein; protein/creatinine ratio; additional laboratory-parameter assessments (e.g., hematocrit, thrombocytes, aspartate aminotransferase, alanine aminotransferase, lactate dehydrogenase, bilirubin [indirect], uric acid, serum creatinine and haptoglobin); and neurologic assessment. 1.) Hund M, Verhagen-Kamerbeek W, Reim M, Messinger D, Van der Does R, Stepan H. Influence of the sFlt-1/PlGF ratio on clinical decision-making in women with suspected preeclampsia—the PreOS study protocol. Hypertens Pregnancy. 2015;34(1):102–115.
(TIFF)

S2 Table Patient populations in the PreOS study. HELLP, hemolysis, elevated liver enzymes and low platelets; PlGF, placental growth factor; PreOS, Preeclampsia Open Study; sFlt-1, soluble fms-like tyrosine kinase 1.
(TIF)

S3 Table Patient enrollment per site. (TIF)

S4 Table Disposition of patients through the study. PlGF, placental growth factor; sFlt-1, soluble fms-like tyrosine kinase 1. * Baseline and interim visits were counted only if the sFlt-1/PlGF ratio was measured and intended procedures before and after knowledge of sFlt-1/PlGF ratio were at least partly documented. ^†^ Delivery visits were counted only if the date of delivery was available. ^‡^ Postpartum visits were counted only if at least one of the scheduled assessments was partly done.
(TIF)

S5 Table Reasons for exclusion from the PreOS study populations (more than one reason for exclusion may apply to each patient). BP, blood pressure; HELLP, hemolysis, elevated liver enzymes and low platelet count; PlGF, placental growth factor; PreOS, Preeclampsia Open Study; sFlt-1, soluble fms-like tyrosine kinase.
(TIF)

S6 Table Multiple logistic regression analysis (sFlt-1/PlGF ratio and outcome) (full analysis population, n = 192). CI, confidence interval; HELLP, hemolysis, elevated liver enzymes and low platelets; IUGR, intrauterine growth restriction; PlGF, placental growth factor; RDS, respiratory distress syndrome; sFlt-1, soluble fms-like tyrosine kinase 1.
(TIF)

S7 Table Intended admission to hospital before and after knowledge of the sFlt-1/PlGF ratio (per-protocol population, n = 118). PlGF, placental growth factor; sFlt-1, soluble fms-like tyrosine kinase 1.
(TIF)
