# Supplementary figures and images for: Influence of the sFlt-1/PlGF Ratio on Clinical Decision-Making in Women with Suspected Preeclampsia
Source: PLoS One. 2016 May 31;11(5):e0156013. doi: 10.1371/journal.pone.0156013 (PMC4887119; doi:10.1371/journal.pone.0156013)

**S1 Fig. iPad® application used in PreOS.**


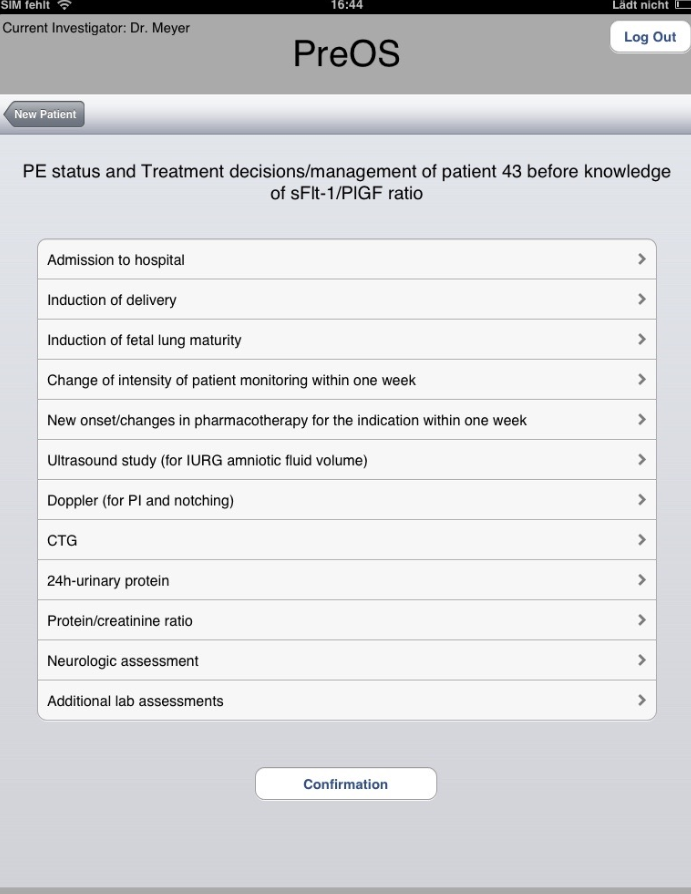


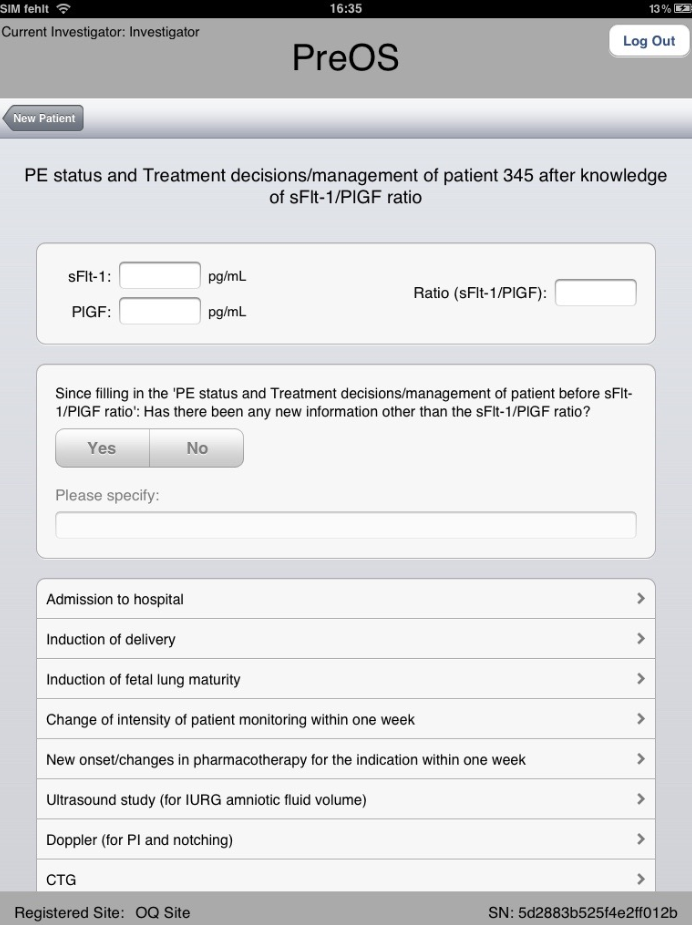


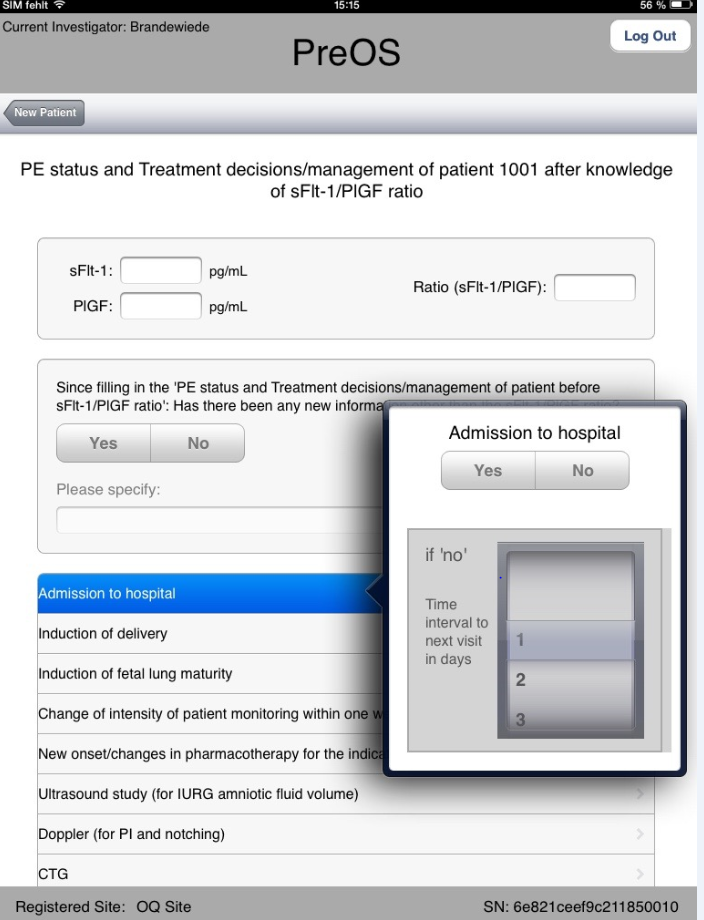

Supplement: S1 Fig — (DOCX) [file pone.0156013.s001.docx]

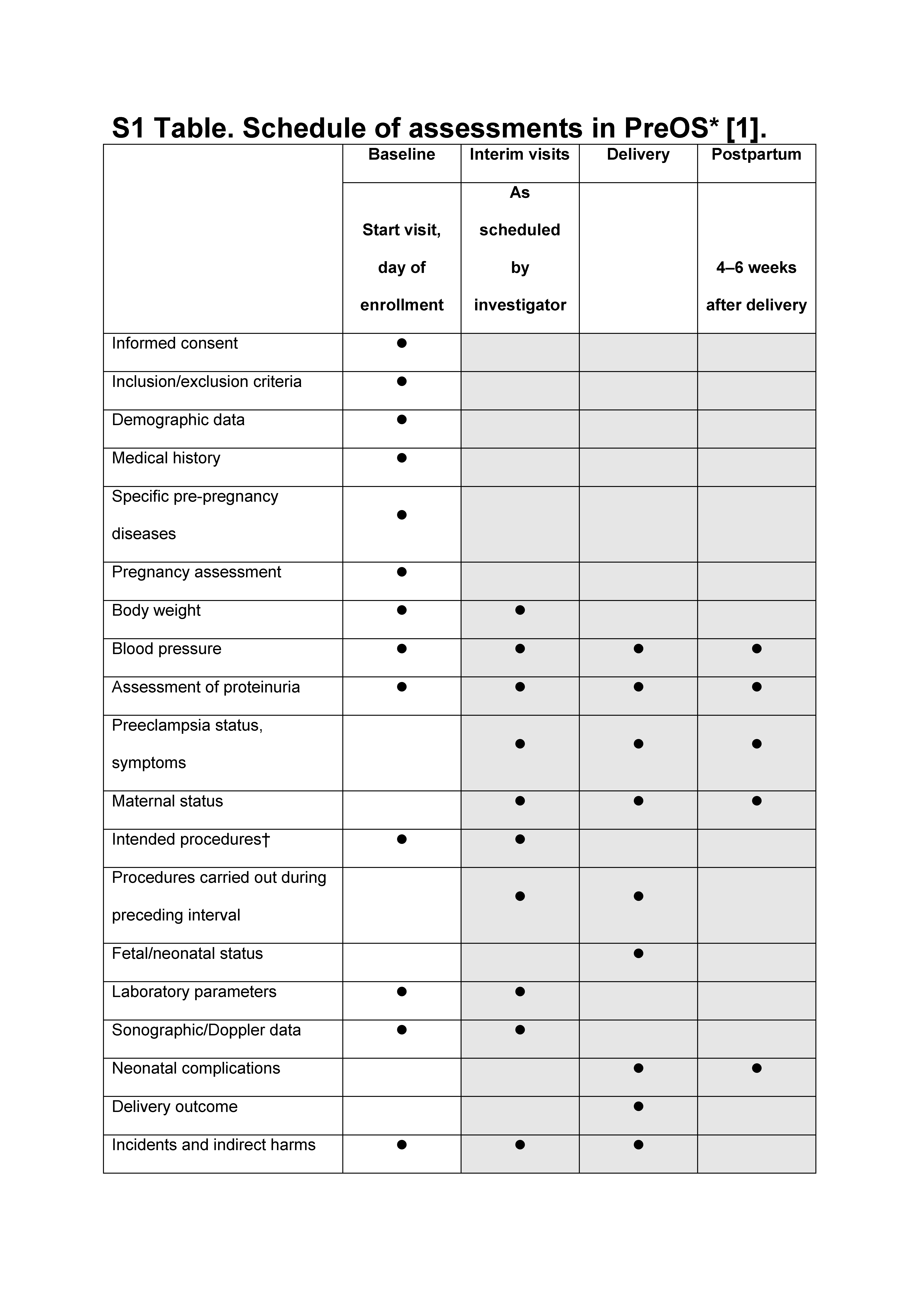

Supplement: S1 Table — IUGR, intrauterine growth restriction; PlGF, placental growth factor; PreOS, Preeclampsia Open Study1; sFlt-1, soluble fms-like tyrosine kinase 1.* Measures listed not stipulated by the protocol. Documentation of data as available based on routine proceedings at the center and clinical judgment. † Intended procedures included: admission to hospital; induction of delivery; induction of fetal lung maturity; time interval to next visit; change in intensity of patient monitoring (intervals of blood pressure measurement; blood pressure home monitoring); assessment of protein in urine; new onset or changes in (pharmaco-)therapy for the indication; ultrasound study (for IUGR, amniotic fluid volume) and/or uterine artery Doppler (for pulsatility index notching); cardiotocography; 24-hour urinary protein; protein/creatinine ratio; additional laboratory-parameter assessments (e.g., hematocrit, thrombocytes, aspartate aminotransferase, alanine aminotransferase, lactate dehydrogenase, bilirubin [indirect], uric acid, serum creatinine and haptoglobin); and neurologic assessment. 1.) Hund M, Verhagen-Kamerbeek W, Reim M, Messinger D, Van der Does R, Stepan H. Influence of the sFlt-1/PlGF ratio on clinical decision-making in women with suspected preeclampsia—the PreOS study protocol. Hypertens Pregnancy. 2015;34(1):102–115. (TIFF) [file pone.0156013.s002.tiff]
